# Quinolinic Acid: An Endogenous Neurotoxin with Multiple Targets

**DOI:** 10.1155/2013/104024

**Published:** 2013-09-05

**Authors:** Rafael Lugo-Huitrón, Perla Ugalde Muñiz, Benjamin Pineda, José Pedraza-Chaverrí, Camilo Ríos, Verónica Pérez-de la Cruz

**Affiliations:** ^1^Departamento de Neuroquímica, Instituto Nacional de Neurología y Neurocirugía Manuel Velasco Suárez, Insurgentes Sur 3877, S.S.A., 14269 México, DF, Mexico; ^2^Laboratorio de Neuroinmunología, Instituto Nacional de Neurología y Neurocirugía Manuel Velasco Suárez, Insurgentes Sur 3877, S.S.A., 14269 México, DF, Mexico; ^3^Departamento de Biología, Facultad de Química, Universidad Nacional Autónoma de México, 04510 México, DF, Mexico

## Abstract

Quinolinic acid (QUIN), a neuroactive metabolite of the kynurenine pathway, is normally presented in nanomolar concentrations in human brain and cerebrospinal fluid (CSF) and is often implicated in the pathogenesis of a variety of human neurological diseases. QUIN is an agonist of N-methyl-D-aspartate (NMDA) receptor, and it has a high *in vivo* potency as an excitotoxin. In fact, although QUIN has an uptake system, its neuronal degradation enzyme is rapidly saturated, and the rest of extracellular QUIN can continue stimulating the NMDA receptor. However, its toxicity cannot be fully explained by its activation of NMDA receptors it is likely that additional mechanisms may also be involved. In this review we describe some of the most relevant targets of QUIN neurotoxicity which involves presynaptic receptors, energetic dysfunction, oxidative stress, transcription factors, cytoskeletal disruption, behavior alterations, and cell death.

## 1. Biosynthesis of Quinolinic Acid (QUIN)

**Figure 1:**
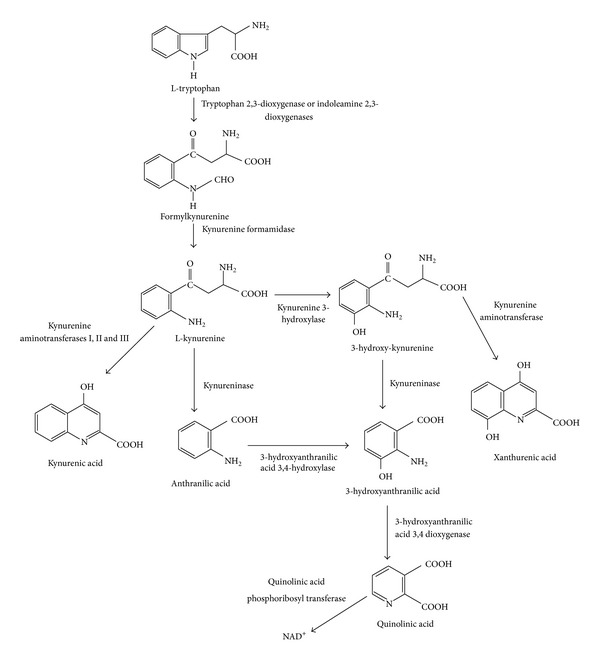
Kynurenine pathway. NAD^+^= nicotinamide adenine dinucleotide.

Tryptophan (TRP) is an essential amino acid that has various important biological functions. In mammals, about 90% of dietary TRP is metabolized along the kynurenine pathway (KP) (Figure 1) [1, 2], which represents the major catabolic route of TRP and a source of nicotinamide adenine nucleotide (NAD^+^), a cofactor in cellular respiration and energy production that plays an important role in the DNA repair and transcriptional regulation [3, 4]. In recent years, the KP has been studied given that it contains metabolites with neuroactive and redox properties. An imbalance in the levels of some metabolites of this pathway has been involved in different pathologies. 

The first regulatory step of the KP is the oxidative cleavage of the TRP by tryptophan 2,3-dioxygenase and indolamine 2,3-dioxygenases 1 and 2 (IDO-1 and IDO-2). The product of this cleavage is formylkynurenine, which is hydrolyzed by a formamidase enzyme to give kynurenine (KYN). This metabolite is at a branch point in the pathway and can be further metabolized by three different enzymes: (1) kynureninase, which catalyzes the conversion of KYN to anthranilic acid (AA), (2) kynurenine aminotransferases I, II and III, which catalyze the transamination of KYN to form kynurenic acid (KYNA), and (3) kynurenine 3-hydroxylase, which produces 3-hydroxykynurenine (3-HK) from L-KYN. This branch is the most important route for QUIN synthesis, and it is known that this enzyme has the highest affinity for L-KYN, suggesting that under normal conditions, it metabolizes most of the available kynurenine [5]. At this point, kynureninase cleaves the 3-HK to give 3-hydroxyanthranilic acid (3-HA). The 3-hydroxyanthranilic acid oxygenase (3-HAO) catalyzes the conversion of 3-HA acid to an unstable intermediate, aminocarboxymuconic semialdehyde, which then preferentially converts to QUIN by a nonenzymatic cyclisation [6]. This intermediate compound can also produce picolinic acid instead of QUIN [7]. 3-HAO is an iron dependent enzyme requiring Fe^2+^ ions and sulfhydryl groups for its activity and is presented in the mitochondrial membrane [8] and in the excitatory synapses [9]. Finally, QUIN is catabolized to NAD^+^ and carbon dioxide by the action of quinolinate phosphoribosyl transferase (QPRT). This enzyme has been identified in rat and human CNS tissue [10]. Magnesium ions are required for QPRT activity and there is evidence that a cysteine residue at the active site is required for catalysis [11]. Interestingly, a different brain localization of 3-HAO and QPRT has been observed, while 3-HAO is in the soluble fraction of brain homogenate, QPRT is in a P2 synaptosomal fractions particulate component [12]. For this reason, QUIN is produced by microglia [13–15] and must exit those cells to be metabolized by QPRT in a separate population of QPRT-containing astrocytes and neurons [15].

The concentration of QUIN varies among different brain regions, with the cerebral cortex containing approximately 1.8 nmol/g wet weight; almost 2-fold than that found in the hippocampus (1 nmol/g wet weight) [16–18].

## 2. Metabolism of QUIN 

Intraarterial administration of either micromolar or millimolar concentrations of QUIN resulted in only negligible accumulations of this metabolite in the brain, suggesting that the central nervous system (CNS) appears to be well protected by the blood brain barrier (BBB) from peripheral QUIN [19].

Many factors come into play to render QUIN a potent neurotoxin. One of such factors is the performance of the two enzymes involved in QUIN synthesis and metabolism, respectively. There are substantially fewer cells containing QPRT than those that contain 3-HAO [20]. The brain area with the highest QPRT activity is the olfactory bulb, and among the regions with the lowest activity are the frontal cortex, striatum, hippocampus, and retina [11]. A detailed analysis of the properties of 3-HAO and QPRT indicates that both have similar Km values, but 3-HAO reaction velocity was 80-fold higher than QPRT [21]. Consequently, the production of QUIN occurs at a much faster rate within the brain than the conversion to NAD^+^. This has implications for the accumulation of QUIN in the brain under certain pathological conditions. Furthermore, 3-HAO activity may normally be restrained by factors such as the availability of Fe^2+^ ions [8, 22, 23]. Stachowski and Schwarcz [23], showed that Fe is a cofactor of 3-HAO and the addition of Fe^2+^ (2–40 *μ*M) stimulated 3-HAO activity 4- to 6-fold in striatal homogenates of mouse, rat, and human; this effect was prevented by ferritin. Thus, if in neuronal damage occurs releasing of Fe^2+^ ions, which is normally the case, then the production of QUIN would be elevated, thereby causing more damage and so the vicious circle would continue. 

Some years ago, studies with [^3^H]QUIN in hippocampus showed that this region as well as the striatum does not appear to possess mechanisms either for the rapid removal of QUIN or for its metabolic degradation in the extracellular space by QPRT [19]. Recently, it was shown that human-mixed brain cells (neurons, astrocytes, and microglia) can take large amounts of QUIN and saturate the neuronal QPRT; however, the precise mechanism by which QUIN is taken up by neurons and astrocytes is unclear [24–26]. All these factors promote QUIN's ability to cause cellular damage.

Additionally, the concentration and metabolism of QUIN appears to depend on the age of the rat. In fact, Moroni and coworkers [17] found that the administration of TRP was able to increase QUIN levels in adult rats but not in newborn rats. Furthermore, a progressive QUIN increase was found in rats of 3 days and 3, 9, and 30-months of age. In rats of 30 month old, half of them were found to have QUIN concentrations approaching to those that cause neurotoxicity [27]. 

On the other hand, the administration of a TRP-free diet to rats for 15 days resulted in a doubling of QUIN concentrations in the cortex. One explanation for this may be that QUIN can also be synthesized by a different pathway, particularly as some bacteria and plants are able to synthesize QUIN from the condensation of aspartic acid and dihydroxyacetate [8].

## 3. Excitotoxicity Produced by QUIN

The first evidence that kynurenines may have a role in brain function was given by Lapin [28], who observed convulsions in mice after an intracerebroventricular QUIN injection. In 1981, Stone and Perkins discovered that QUIN was a potent excitant of neurons in the CNS, by acting as an agonist at the *N*-methyl-*D*-aspartate (NMDA) sensitive population glutamate receptors [29], and Schwarcz and coworkers (1983) were the first who demonstrated that QUIN causes selective neuronal lesions, and they also found that focal injections of QUIN into the striatum resulted in neurochemical, behavioral and pathological changes [30].

In the 80's, it was demonstrated that QUIN is about one-quarter as active as NMDA and approximately as active as glutamate and aspartate at stimulating NMDA receptors [29]. It must however be remembered that the latter compounds have an rapid, high-affinity uptake system for their removal from the synapse, while QUIN has a uptake system, but the neuronal QPRT is rapidly saturated by this metabolite (~300 nM) [25]. Although part of QUIN can be removed from the synaptic cleft, the rest of QUIN will continue stimulating the NMDA receptor causing extensive damage. QUIN acts selectively at NMDA receptors, specifically with NMDA receptor subtypes containing the NR2A and NR2B subunits [31], with massive calcium entry into neurons and astrocytes. Therefore, QUIN exerts the greatest damage to neurons where these receptor subtypes are present. Areas of the brain most sensitive to QUIN neurotoxicity are the hippocampus and striatum [32] in which the NMDA receptors are widely distributed [33]. Within these brain areas, some neuronal cell types are more sensitive than others, with cholinergic neuronal death in the striatum observed following QUIN injection [34] and preferential susceptibility of pyramidal cells in the hippocampus [32]. Striatal spiny neurons containing the neurotransmitter *γ*-aminobutyric acid (GABA) and substance P are also sensitive to QUIN toxicity, with the subclass of striatal spiny neurons containing somatostatin and neuropeptide Y being preserved [35]. 

QUIN can also increase glutamate release and inhibit its reuptake by astrocytes, thus increasing its concentration in the microenvironments, causing neurotoxicity [36, 37] and also limiting glutamate to glutamine recycling in astrocytes by decreasing glutamine synthetase activity [38, 39]. As shown previously in cortical neurons, dopaminergic neurons do not produce QUIN but take it up from the microenvironment [15, 40]. On the other hand, QUIN (10 *μ*M) prevents of glutamate-induced excitotoxicity in primary cultures of rat cerebellar granule neurons [41], nevertheless mature organotypic cultures of rat corticostriatal system or caudate nucleus chronically exposed to 100 nM QUIN for up to 7 weeks show focal degeneration characterized by the presence of vacuoles in neuropil, swollen dendrites, occasional swollen post-synaptic elements, and degenerated neurons [42, 43].

Additionally, chronic exposure of human neurons to QUIN causes significant structural changes including dendritic beading, microtubular disruption, and a decrease in organelles. Rahman and coworkers show that the *in vitro* QUIN treatment of human primary foetal neurons led to a substantial increase of tau phosphorylation at multiple positions. The observed increase in QUIN-induced phosphorylation of tau was attributed to a decrease in the expression and activity of the major tau phosphatases [25]. 

Recently, Pierozan and coworkers (2010) described that acute intrastriatal administration of QUIN targets the phosphorylating system associated with the cytoskeleton of neural striatal cells, causing intermediate filament hyperphosphorylation; this effect was mediated by Ca^2+^ influx through NMDA channels and by oxidative stress [44]. Additionally, alterations in the homeostasis of the cytoskeleton of astrocytes and neurons were found in rat striatal slices treated with 100 *μ*M of QUIN. These events were secondary to the following specific mechanism: (a) in astrocytes, the effect by QUIN was mediated by increased Ca^2+^ influx through NMDA receptors and L-type voltage-dependent Ca^2+^ channels (L-VDCC) and (b) in neurons, QUIN actions involving metabotropic glutamate receptors and the Ca^2+^ from intracellular stores besides Ca^2+^ influx through NMDA receptor and L-VDCC. In both cases the increase in the intracellular Ca^2+^ levels set off a cascade of events including activation of the second messengers-dependent protein kinases, which phosphorylate head domain sites on GFAP and neurofilaments subunits and potentially misregulating intermediate filament assembly in both glia and neuronal cells [45]. Additionally, the *in vivo* overstimulation of NMDA receptors by QUIN causes an early impairment of the sarco/endoplasmic reticulum Ca^2+^-ATPase (SERCA) pump which may result in important disturbances in intracellular Ca^2+^ signaling [46].

## 4. Alterations Energetic and QUIN

Recent evidence shows that metabolic impairment is an important mechanism by which QUIN can exert its toxicity. In this context, it was found that QUIN can inhibit B monoamine oxidase (MAO-B) in human brain synaptosomal mitochondria [47] and also can be a potent inhibitor of phosphoenolpyruvate carboxykinase (EC 4.1.1.32) from rat liver cytoplasm, an important enzyme in the gluconeogenesis pathway that converts oxaloacetate to phosphoenolpyruvate [12]. QUIN can potentiate its own toxicity and that of other excitotoxins, like NMDA and glutamate, producing progressive mitochondrial dysfunction [48].

Different studies have shown that intrastriatal injection of QUIN provokes a decrease of cellular respiration and ATP levels [48, 49]; however, these findings may be due to a primary activation of glutamate receptors and a secondary effect of QUIN on energy production via free radicals [50–52]. However, Ribeiro and coworkers [53] observed that QUIN injection also inhibited creatine kinase activity, an important enzyme involved in intracellular energy transfer. QUIN also provoked significant reductions of the activities of complexes II (50%), II–III (35%), and III (46%) of the respiratory chain in the striatum of young rats, and this impairment of striatum bioenergetics induced by QUIN injection was partially mediated by generation of reactive species.

 Recently, Schuck and coworkers [54] have shown that QUIN inhibits the ^14^CO_2_ production and increased glucose uptake in cerebral cortex homogenates of young rats indicating that this kynurenine stimulated the transport and/or utilization of this substrate by the brain. QUIN also inhibits around 35% succinate dehydrogenase (SDH), an enzyme involved in the citric acid cycle and in the respiratory chain. Moreover, this effect was not dependent of the NMDA receptor since MK-801 and kynurenic acid (two NMDA receptor antagonists) and L-NG-nitroarginine methyl ester (L-NAME), a nitric oxide synthase (NOS) inhibitor, did not prevent the inhibition, but the preincubation with superoxide dismutase and catalase can do it. In this context, our group has been shown that QUIN interacts in the SDH-binding site with the arginine 297 residue (R290 of the sequence numbering of SDH-QUIN by docking), whose positive charge is important for the binding affinity of negatively charged inhibitors, and in *in vitro* assays QUIN can inhibit SDH and ATP levels, and the effect in the enzyme is depending on each brain region in which mitochondria were isolated [55]. All this evidence suggests that QUIN has different targets that could be independent of its agonist activity under NMDA receptor, and the mitochondrial impairment represent other mechanism of the QUIN toxicity.

## 5. Oxidative Stress and QUIN

Free radical generation and oxidative stress are involved in the QUIN-induced toxicity; however, we need to take in mind that these mechanisms can be dependent and/or independent of its activity on NMDA receptors. In this line, it has been shown that QUIN can produce oxidative damage independent of its activity under NMDA receptor; this mechanism involves a complex between QUIN and Fe^2+^. Studies by Stipêk and coworkers [62] showed that the lipid peroxidation induced by QUIN was modulated by its interaction with Fe^2+^ to form QUIN-Fe^2+^ complexes that mediate reactive oxygen species (ROS) generation. In phosphate buffer, the QUIN-Fe^2+^ enhanced the formation of the hydroxyl radical via the Fenton reaction [105], and it was also observed that QUIN inhibits the autooxidation of Fe^2+^ by the complex formation. The QUIN-Fe^2+^ complex was shown to be responsible for the *in vitro* DNA chain breakage and lipid peroxidation mediated by hydroxyl radicals [106]. 

Moreover, there is evidence showing that QUIN can increase free radical production by inducing NOS activity in astrocytes and neurons, leading to oxidative stress, increasing both poly(ADP-ribose) polymerase (PARP) activity and extracellular lactate dehydrogenase (LDH) activity [90]. In concordance, striatal slices exposed to QUIN show an increase in both lipid peroxidation and LDH activity and a decrease in mitochondrial function [107]; these alterations were related with proteases activation. 

Furthermore, it has been shown the QUIN capacity to modify the profiles of some endogenous antioxidants in rat brain such as the content of reduced glutathione and copper and zinc-dependent superoxide dismutase activity (Cu, Zn-SOD) [108] and its ability to generate during early stages of toxicity ^•^OH radical [109] and peroxynitrite [86] and to increase lipid peroxidation [108, 110]. In rat brain, intracerebral injection of QUIN resulted in significant neuronal loss and a markedly increased level of SOD1 expression in a time-dependent manner [111]; this increase in SOD1 expression was thought to be a neuroprotective response to limit the oxidative damage caused by QUIN. In support of these results, it was found that QUIN infusion induced cell damage an increase in ROS levels in mice hippocampus, while the second one became normal after 24 hours, the first one persisted for 72 hours. Therefore, the delayed and persistent increase in the antioxidant capacity after QUIN insult may be a cellular adaptive response, probably contributing to the decrease in ROS levels [52]. Additionally, it was observed in synaptosomal fractions exposed to QUIN and 3-nitropropionic acid at nontoxic concentration, a synergic effect in oxidative markers which was just partially prevented by MK-801 [79]. 

Recently, Tronel and coworkers [112] showed that the HO-1 inducer hemin had a deleterious effect in QUIN *in vivo* model and enhanced tissue loss and microglia activation and showed that this effect is probably linked to a hyperproduction of ROS and iron accumulation.

Different ROS scavengers, molecules with antioxidant properties, inducers of activity of antioxidant enzymes, and others drugs have been tested successfully against QUIN toxicity (Table 1), indicating the importance of oxidative damage in the neurodegeneration induced by QUIN.

Based on this evidence and considering that oxidative stress results from an unbalance between the antioxidant defense and the reactive species formed, this phenomenon should be considered as one of the many mechanism by which QUIN exerts its toxic effect since free radicals also can activate more signaling cascades that can contribute and maximize its neurotoxic effect.

## 6. Inflammation and QUIN

Inflammatory events are also implicated in the QUIN toxicity. It is known that microglia is responsible for inflammatory responses in the CNS and takes the major role in altered levels of QUIN, since it has been shown that IFN-*γ* and bacterial lipopolysaccharide (LPS) induce IDO and increase QUIN production [15, 113, 114]. This effect could be potentiated by astrocytes since QUIN induces astrogliosis and in consequence the expression and release of cytokines enhancing the inflammatory response that could compromise cell viability [39, 115, 116]. In this regard, in the brain, large amounts of QUIN are produced and secreted by activated microglia [93]. During CNS inflammation, QUIN levels increase in brain homogenate (246-fold) and extracellular fluid (66-fold), mostly due the increase in local QUIN's synthesis rate [117].

On the other hand, it has been reported the influence of QUIN on inflammatory response. The intrastriatal QUIN administration induces a marked expression of tumor necrosis alpha (TNF-*α*) [118] and interleukin-6 (IL-6) [119] that can be attenuated by inhibiting the cyclooxygenase 2 (COX-2) [120]. QUIN and TNF-*α* cause oligodendrocyte death by apoptotic process [121, 122]. Although TNF-*α* is not neurotoxic itself, this proinflammatory cytokine can contribute to neuronal damage through a variety of effects, such as stimulation of free radical formation, induction of cellular adhesion molecule expression, or potentiation of glutamate-mediate neurotoxicity [123, 124]. Besides, QUIN was able to increase MCP-1 production [116] in human fetal astrocytes, and this effect is likely to be biologically significant. In fact, within the brain, MCP-1 is probably one of the most powerful chemoattractants for monocytes and is mainly produced by astrocytes [125]. During pathological events, macrophages in blood stream also contribute to QUIN formation after an inflammatory response, and this may break blood brain barrier and release QUIN into the brain [126]. In this context, it has been reported that macrophages have the ability to produce approximately 20- to 30-fold more QUIN than microglia [14]. This fact suggests the prevalence of positive feedback in which inflammatory (local or systemic) responses given by microglia or macrophages, respectively, increase QUIN synthesis, and this effect could induce expression of cytokines where both factors may converge resulting in cell death. In this line, Erhardt and coworkers [99] found significantly increased levels of QUIN in the CSF of suicide attempters, and there was a significant correlation between CSF levels of QUIN and the proinflammatory cytokine IL-6. 

## 7. Behavioral, Morphological Alterations and Death Induced by QUIN

Several reports have been shown that the intrastriatal administration of QUIN in rats produced significant behavioral changes. Intrastriatal rat injection of QUIN resulted in an initial period of involuntary movements and intrahippocampal injection triggering convulsions [34]. Sanberg and coworkers (1989) showed that after 4 weeks after lesion with QUIN (150 and 225 nmol), the rats display significantly increased levels in locomotion, and there was a persistent hyperactivity throughout the nocturnal period [127]. However the bilateral intrastriatal injections with QUIN (120 nmoles per side) produce significant motor/kinetic deficits. The motor alterations were seen at both early (24 h after lesion) and late (7 days after lesion) and comprised total distance walked/traveled—which is probably the most accurate index of motility among all tested here—and vertical activity—likely indicating exploratory behavior [128].

On the other hand, the few studies in which it has been investigated the cognitive deficits of rats with QUIN lesions have indicated that this metabolite causes deficits in spatial reference memory. QUIN disrupted the performance of rats on the radial arm water maze, balance-beam, and open-field tasks [129]. In rodents, QUIN unilateral lesion with asymmetrical rotation behavior occurs stimulated by apomorphine, a widely known dopamine agonist [130]. The rotation behavior results from an imbalance of dopaminergic signaling between the injured and the intact hemisphere.

Administration of QUIN directly into the rat striatum produced “axon-sparing” lesions, with marked swelling of dendrites and loss of cell structure in postsynaptic sites, but generally good preservation of axons and presynaptic terminals [30, 131]. Infusion of 120 nmol QUIN into several regions of the rat's brain revealed differences in vulnerability to its neurotoxic effects. The striatum, the pallidal formation, and the hippocampus were the most susceptive brain areas whereas cerebellum, substantia nigra, amygdala, medial septum, and hypothalamus were more resistant [32]. Within these brain areas, some neuronal cell types are more sensitive than others, with cholinergic neuronal death in the striatum observed following QUIN and preferential susceptibility of pyramidal cells in the hippocampus [32]. Striatal spiny neurons containing the neurotransmitter *γ*-aminobutyric acid (GABA) and substance P are also sensitive to QUIN toxicity, with the subclass of striatal spiny neurons containing somatostatin and neuropeptide Y being preserved [35]. Moreover, intrastriatal injections of QUIN cause significant striatal atrophy, ventricular dilation, metabolic depression, and loss of neurons in the striatum. Histological evaluation of cytochrome-oxidase-stained tissue indicated that intrastriatal injections of QUIN caused widespread metabolic depression and QUIN (200 nmoles) results in relatively extensive loss of NADPH-diaphorase-containing neurons [129]. QUIN induces not only cell death, but also damage to axons and dendrites [43, 132]; in this regard, recent studies demonstrated that QUIN toxicity could lead to destabilization of the cytoskeleton by phosphorylating structural proteins [25, 44]. The cytoskeleton plays a key role in maintaining the neuronal cell shape and is essential for its normal functions, such as neurite outgrowth, synapse formation, and internal transport of various molecules.

 QUIN resulted in neurons that displayed a nonapoptotic pattern of chromatin condensation and early disruption of cytoplasmic organelles. QUIN-injured neurons underwent changes in mitochondria and endoplasmic reticulum [133]. It was also demonstrated that QPRT-depleted cells had an increased intracellular active-caspase-3 activity and were highly sensitive to spontaneous cell death [134].

QUIN has been demonstrated to induce neuronal and astrocytic apoptosis involving the activation of caspase 3 [135–137]. Another study demonstrated that intrastriatal injection of QUIN in rat brain leads to the hyperphosphorylation of cytoskeletal intermediate filament proteins in astrocytes and neurons [44]. It has been shown that intrastriatal injection of different doses of QUIN causes apoptotic cell death [138–140], and in striatal cells, this kind of death is mediated by an increase in Bax and a decrease in Bcl-2 protein levels, leading to reduced levels of Bax:Bcl-2 heterodimers [141]. In fact, Bcl-2 and Bcl-xL protein levels were downregulated at later times after QUIN injection suggesting that apoptotic cell death may, in part, be related to reduced levels of antiapoptotic proteins [141]. Besides striatal NMDA receptor stimulation by QUIN promotes the selective degradation of I*κ*B-*α*, this degradation appears to be mediated by caspase-3-like protease and promotes an apoptotic response that involves the NF-*κ*B activation [142]. Recently, it was shown that during the process of neuronal cell death induced by QUIN, upregulation of p53 and proapoptotic p53 target genes PUMA (p53-upregulated modulator of apoptosis) and Bax and downregulation of antiapoptotic protein Bcl-2 were observed. Moreover, QUIN induced the expression of damage-regulated autophagy modulator (DRAM), beclin 1, and LC3-II, proteins that are involved in autophagy [143]. All this evidence suggests that NF-*κ*B-dependent p53 induction contributes to QUIN-induced death of striatal neurons through both apoptotic and autophagic mechanisms.

## 8. QUIN and Neurodegenerative Disease

It is known that kynurenine pathway is found in glial cells of the CNS and in inflammatory cells of the circulation, and it is regulated by redox components as well as by inflammatory components. The fact that different neuropathologies present excitotoxicity, oxidative stress, and inflammation as common factors, suggests that KP metabolites may be altered. In this context, different groups have been shown that in some brain pathologies as well as in experimental models of neurodegeneration, an inappropriate activation of KP may lead to increased QUIN levels. Alteration in QUIN levels has been implicated in different pathologies such as Alzheimer's, Huntington's, and Parkinson's diseases as well as in experimental models of those diseases in which QUIN plays a special role acting on the neurodegenerative cascade (Table 2).

## 9. Conclusion

**Figure 2:**
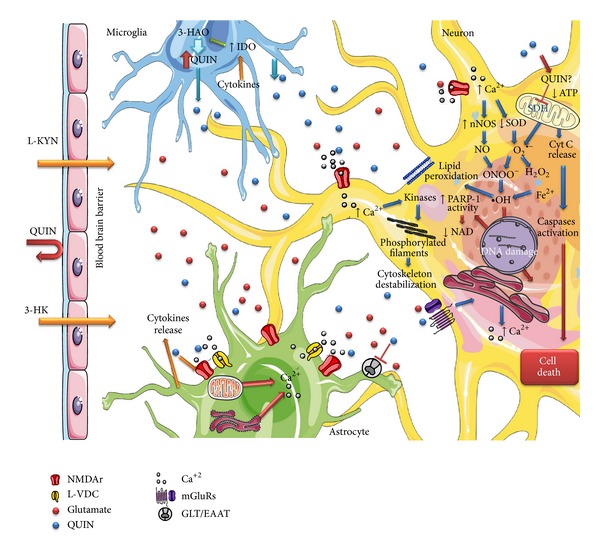
Multiple mechanisms leading to QUIN cytotoxicity. One of the principal toxicity mechanism of QUIN is through the over stimulation of the NMDA receptor which is powered by the lack of uptake of QUIN from the extracellular space. Additionally, QUIN enhances the release of synaptosomal glutamate as a consequence of the inhibition of glutamate uptake into the astrocytes that will lead to overstimulation of receptors. Furthermore, QUIN can decrease the activity of antioxidant enzymes promoting ROS production and generating lipid peroxidation. Also, QUIN may inhibit the activity of mitochondrial complexes leading to energetic deficit, activating caspases and releasing cytochrome c. All these factors induce cytoskeleton destabilization, DNA damage, and cell death.

According to the information that has been reviewed, the mechanisms by which QUIN produces neurotoxicity include overactivation of the NMDA receptor, energy deficit, oxidative stress, and cell death. A sequence of these events is described in Figure 2. Far from being excluding, all these factors are somehow closely related and also act synergistically to induce neurodegeneration. Taking into account that QUIN has been implicated in neurodegenerative diseases and some of their toxic mechanisms are still unknown, the challenges for the future research should be directed to clarify all the possible routes that can promote or contribute to the damage induced by this metabolite. This may help to explain the physiopathological events occurring in several neurodegenerative diseases in which the levels of QUIN are increased.

## Figures and Tables

**Table 1:** Effect of various molecules on the toxicity induced by QUIN.

Compound	Mechanism of action	Reference
Melatonin	(i) Attenuates the convulsant effect of quinolinate (ii) Partially protects against the increase of circling behavior (iii) Protects against the increase in ROS and protein carbonyl levels as well as the inhibition of superoxide dismutase activity	[56–58]

Denepryl	(i) Acts as a potent-free radical scavenger (ii) Increases the activity of endogenous antioxidant enzymes	[59]

*α*-phenyl-t-butyl nitrone	(i) Presents cytoprotective effects	[60]

Polyamines such as spermine and spermidine	(i) Inhibit QUIN-induced TBARS production and have antioxidant properties	[61]

Deferoxamine (iron chelator)	(i) Reduces lipid peroxidation	[62]

Reduced glutathione	(i) Decreases lipid peroxidation and ROS formation in brain synaptosomes	[63]

Selenium	(i) Attenuates the QUIN-induced early reactive oxygen species formation and lipid peroxidation (ii) Prevents loss of mitochondrial reductive capacity and morphological alterations in the striatum (iii) Induces stimulation of striatal GPx activity (iv) Prevents I*κ*B-*α* degradation (v) Reduces the nuclear translocation of NF-*κ*B and inhibits the activity of caspase-3, resulting in internucleosomal DNA preservation (vi) Induces an early stimulation of TrxR activity	[64–66]

Selenocompounds such as ebselen	(i) Inhibit TBARS production	[67]

Ksheerabala	(i) Decreases de lipid peroxidation and protein peroxidation (ii) Increases the activity of antioxidant enzymes	[68]

Licofelone, Montelukast, and Pioglitazone	(i) Significantly improve body weight, locomotor activity, oxidative defense, activity of mitochondrial enzyme complex, rotarod performance, and balance beam walk	[69–71]

Dizocilpine (MK-801)	(i) Improves body weight, behavioral alterations (locomotor activity and rotarod performance) and attenuates oxidative damage and mitochondrial enzymes complexes dysfunction (ii) Improves learning task in rats receiving chronic i.c.v, infusion of QUIN (iii) Decreases release of lactate dehydrogenase (LDH) in NSC-34 cells after 48 h of QUIN	[72–74]

Nimesulide, rofecoxib, and caffeic acid	(i) Restore mitochondrial enzyme complex activities in striatum	[75, 76]

Memantine	(i) Significantly attenuates QUIN-mediated PARP activation, NAD^+^ depletion, and LDH release in both neurons and astrocytes as well as decreases LDH release in NSC-34 cells induced by QUIN	[74, 77]

2-amino-5-phosphonopentanoic acid (APV)	(i) Decreases QUIN-induced LDH release in NSC-34 cell (ii) Abolishes the release of aspartate and glutamate	[74, 78]

L-carnitine and acetyl L-carnitine	(i) Reduce lipid peroxidation (ii) Prevent mitochondrial dysfunction in brain synaptosomes (iii) Attenuate the behavioral alterations and striatal degeneration	[79–81]

Tolmetin and sulindac	(i) Reduce the generation of superoxide anions (ii) Reduce the lipid peroxidation after an intrahippocampal injection of QUIN (iii) Reduce the spatial memory deficit	[82, 83]

Acyclovir	(i) Inhibits the lipid peroxidation after *in vitro* and *in vivo* exposure to QUIN (ii) Reduces necrosis of hippocampal neurons	[84]

Nitroarginine and L-arginine	(i) Prevent lipid peroxidation induced by QUIN	[85]

Iron metalloporphyrins such as Fe(TPFPP) and Fe(TPPS)	(i) Decrease 3-nitrotyrosine levels (ii) Prevent lipid peroxidation and mitochondrial dysfunction (iii) Reduce the DNA fragmentation and decreases caspase-3-like activation (iv) Abolish the circling behavior (v) Partially recover GABA levels (vi) Reduce the immunochemical expression of IL-6 and iNOS	[86–88]

Safranal	(i) Inhibits lipid peroxidation (ii) Inhibits oxidative DNA damage (iii) Improves hippocampal antioxidant and thiol redox status	[89]

Polyphenols (epigallocatechin gallate, curcumin)	(i) Inhibit QUIN-induced nNOS activity and subsequent nitrite production (ii) Reduce 3-nitrotyrosine production (iii) Prevent DNA damage and PARP-1 activation (iv) Attenuate QUIN-induced Ca^2+^ influx	[90]

Thiobarbituric acid reactive species: TBARS; reactive oxygen species: ROS; thioredoxin reductase: TrxR; glutathione peroxidase: GPx; nuclear factor-kappaB: NF-*κ*B; quinolinic acid: QUIN; deoxyribonucleic acid: DNA; *γ*-Aminobutyric acid: GABA; interleukin 6: IL-6; inducible nitric oxide synthase: iNOS.

**Table 2:** Alterations in QUIN levels presented in different neurodegenerative diseases and experimental models. Key references are shown in the quarter column.

Disease/model	QUIN levels	Associated alterations	Reference
Alzheimer	↑ in demented patient ↑ in senile plaques	(i) IDO overexpression (ii) ↓ KYNA (iii) QUIN colocalizes with tau in cortical sections (iv) ↑ QUIN/3-HK quotient in plasma	[25, 26, 91, 92]

A*β* 1-42	↑	(i) IDO over-expression in microglia and macrophages	[93]

Huntington	↑	(i) ↑ 3-HK	[94]

Huntingtin transgenic mice	↑ in YAC128 mice, Hdh^Q92^/Hdh^Q111^ knock-in mice	(i) ↑ 3-HK	[95]

Human immunodeficiency virus (HIV)	↑ in CSF and serum of patients	(i) Cytokines release (ii) ↑ IDO (iii) QUIN levels enhanced independent blood brain barrier breakdown	[96–98]

Suicide attempters	↑ in CSF of suicide attempters	(i) An increased QUIN/KYNA quotient	[99]

Depression	↑ QUIN expression in human brain during acute depressive episodes	(i) Abnormal NMDA receptor function	[100]

Autism	↑ in CFS of patients	(i) ↑ biopterin (ii) ↓ neopterin	[101]

Amyotrophic lateral sclerosis	↑ in CSF and serum of patients	(i) ↑ TRP and L-KYN, human leukocyte antigen-DR (ii) IDO over-expression	[102]

Experimental allergic encephalomyelitis, a model of multiple sclerosis	↑ in the spinal cords of rats	(i) ↑ in KMO activity and 3-HK levels	[103, 104]

IDO: indolamine 2,3-dioxigenase, KYNA: kynurenic acid, QUIN: quinolinic acid, L-KYN: L-kynurenine, 3-HK: 3-hydroxykynurenine, TRP: tryptophan, and CSF: cerebrospinal fluid.
